# Comparison of threshold selection methods for microarray gene co-expression matrices

**DOI:** 10.1186/1756-0500-2-240

**Published:** 2009-12-02

**Authors:** Bhavesh R Borate, Elissa J Chesler, Michael A Langston, Arnold M Saxton, Brynn H Voy

**Affiliations:** 1 Genome Science and Technology Program, University of Tennessee, Knoxville, Tennessee, USA; 2 Department of Electrical Engineering and Computer Science, University of Tennessee, Knoxville, Tennessee, USA; 3 Oak Ridge National Laboratory, Systems Genetics Group, Biosciences Division, Oak Ridge, Tennessee, USA; 4 Department of Animal Science, University of Tennessee, Knoxville, Tennessee, USA

## Abstract

**Background:**

Network and clustering analyses of microarray co-expression correlation data often require application of a threshold to discard small correlations, thus reducing computational demands and decreasing the number of uninformative correlations. This study investigated threshold selection in the context of combinatorial network analysis of transcriptome data.

**Findings:**

Six conceptually diverse methods - based on number of maximal cliques, correlation of control spots with expressed genes, top 1% of correlations, spectral graph clustering, Bonferroni correction of p-values, and statistical power - were used to estimate a correlation threshold for three time-series microarray datasets. The validity of thresholds was tested by comparison to thresholds derived from Gene Ontology information. Stability and reliability of the best methods were evaluated with block bootstrapping.

Two threshold methods, number of maximal cliques and spectral graph, used information in the correlation matrix structure and performed well in terms of stability. Comparison to Gene Ontology found thresholds from number of maximal cliques extracted from a co-expression matrix were the most biologically valid. Approaches to improve both methods were suggested.

**Conclusion:**

Threshold selection approaches based on network structure of gene relationships gave thresholds with greater relevance to curated biological relationships than approaches based on statistical pair-wise relationships.

## Introduction

To extract gene networks from microarray data, correlations are often used as a measure of gene co-expression. A typical microarray with 20,000 gene probes will produce 200 million correlations. Correlations below a threshold value, closer to zero, will be less meaningful. Hard and soft threshold approaches have been applied to biological data. Hard thresholds discard gene pairs with correlation below the threshold, while soft thresholds use the correlation value to weight gene network relationships. Zhang and Horvath [1] concluded that soft thresholds based on aggregate, modular relationships between genes gave more robust results, but data reduction by a hard threshold is often essential for computational tractability of graph algorithms.

We focus on relevance networks, created by applying a hard threshold to the gene expression correlation matrix [2], then extracting gene networks. The resulting networks have been well documented in recent literature to yield sets of co-expressed genes [3-5]. Relevance networks are easily converted to graphs, with genes as vertices, only connected by an edge if their correlation is above the threshold. A clique is a sub-graph in which all nodes are connected to each other [6]. A disadvantage of using cliques is the computational requirements, which grow exponentially with number of genes. Thus hard threshold selection is required when performing clique extraction on microarray data.

Current approaches to threshold selection are typically statistically based, and do not fully reflect the connectivity of the data [7]. Methods based on statistical arguments may not necessarily yield biologically significant relationships [3,8].

Some studies used an arbitrary threshold correlation such as 0.80 [9]. Moriyama et al. [10] obtained random correlation distributions for gene pairs by permuting their expression values and defended their choice of threshold based on statistical significance. Lee et al. [11] used the top 1% of correlations (absolute value) to build a co-expression network. Voy et al. [3] used distribution of correlations of genes with buffer spots on the arrays to select a threshold correlation value of 0.875.

However, using connectivity of the data to derive thresholds has been suggested. Langston et al. [12] recommended use of ontological distance, statistical significance and various graph structural attributes to arrive at a correlation threshold. Palla et al. [13] found that a threshold based on clique size was effective at separating networks.

Here two threshold selection methods based on correlation graph structure are compared with common statistically based methods. The graph based methods used spectral properties [14] or number of cliques to select a threshold. Objectives were to compare the various hard threshold methods for validity (retention of biological information), stability, and reliability.

## Methods

### Datasets

Three yeast *S. cerevisiae *time-series datasets were chosen for this study: 31 arrays for Anoxia state [15], 21 arrays for Reoxygenation state [15] and 18 arrays from yeast cultures synchronized using Alpha-factor arrest [16]. Data are available on Gene Expression Omnibus under GSE2246, GSE2267 and GSE22. Extensive GO annotation for *S. cerevisiae *genes influenced the selection. Exploratory data analyses within each dataset using PCA, box plots and pair-wise correlations between arrays found no outlier arrays. Quantile plots showed data were normally distributed, and distribution of correlations among gene expression profiles had the expected bell-shaped curve, so all data were used.

### Software

Software written by Langston and colleagues (University of Tennessee) was used, including Datagen version 1.4a for computing correlations, maximal clique enumeration code version 2.0.1 [17], spectral analysis code [14], and GO Pairwise Similarity analysis code version 1.0. Matrix calculations for spectral graph analysis were carried out in MATLAB 7.0. P-values were calculated in SAS version 9.1 (Cary; NC). Statistical power was calculated using PASS statistical software http://www.ncss.com/pass.html.

### Threshold Estimation

Six conceptually different approaches were evaluated:

1) Numbers of maximal cliques were calculated at each potential correlation threshold, starting at r = 0.99. The threshold was lowered, in steps of 0.01, and number of maximal cliques increased due to greater connections among genes. When clique number increased two times (Maximal Clique-2) or three times (Maximal Clique-3) the previous value, that correlation was chosen as the threshold.

2) For each potential threshold correlation value, spectral graph theory [18] was used to decompose the resulting graph into eigenvalues and eigenvectors, which were used to enumerate spectral clusters [19]. As the potential threshold was incrementally lowered in steps of 0.01, a peak in the number of clusters occurs, and the threshold is chosen to maximize cluster number. Details are in [14].

3) Correlations of control spots with all other genes on the array were calculated, creating a null distribution. The 99th percentile correlation value (absolute value) of this distribution gave the threshold.

4) The top 1% of all correlations (absolute value) among genes was used to estimate a threshold [11]. Correlations were ranked, and the correlation at the 99th percentile was the threshold estimate. Note that the control spot method uses a different subset of correlations (only with control spots), whereas this method uses all correlations among genes.

5) A p-value for every correlation was computed, testing if the correlation was zero (Fisher's z-transformation). Threshold estimate was the correlation value corresponding to the critical Bonferroni p-value, 0.05/number of correlations. This threshold will remove any correlations that are statistically equal to zero.

6) Statistical power calculations were used to find the correlation value that gave an 80% chance of rejecting the null hypothesis, Ho: correlation = 0. Type I error rate in these calculations was Bonferroni-adjusted to correct for multiple testing.

Further details on computing these threshold estimation methods are in the Additional file 1.

### Performance Evaluation

Performance of the threshold estimation methods was evaluated by comparison to a biologically based Gene Ontology threshold. GO data used was gene_ontology_edit.obo.2008-05-01.gz. The biological meaning for each correlation bin (in 0.01 increments) was the average of functional similarity scores for all gene pairs within that correlation bin. Functional similarity for a pair of genes was defined as log(n/N)/log(2/N), where n is the number of genes in the lowest GO category that contained both genes, and N is the total number of genes annotated for the organism. The formula normalizes Functional similarity to a 0 to 1 range, and a value of 1 means the GO category contained only the two genes being considered (perfect similarity). GO threshold estimate was defined as the correlation at which change in average functional similarity exceeded median change plus half its standard deviation, thus identifying where biological information begins to accumulate.

To study stability of the methods, 10,000 block bootstrap samples were created by sampling arrays with replacement from each block. Blocks were defined to be 2 or 3 adjacent time periods, such that each block contained 3 or 4 arrays. Block bootstrapping was necessary to preserve as much as possible the time-course dependency structure of the experiments [20]. For each of the 10,000 samples, a threshold estimate was calculated by each method, and the distribution of these thresholds was used to compare threshold methods for stability.

## Results

**Figure 1:**
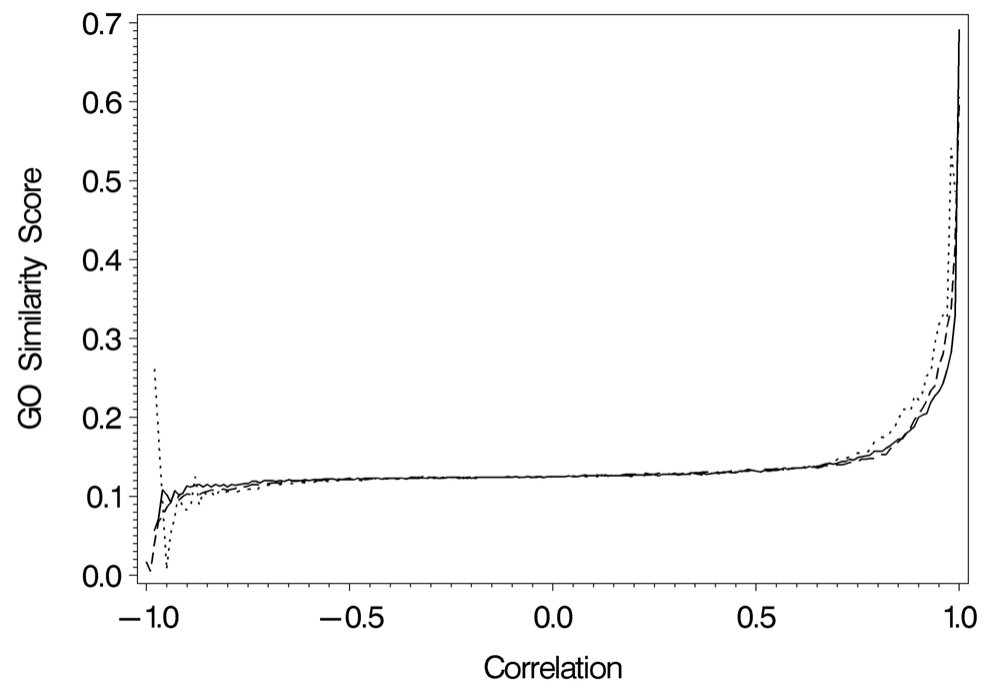
**Change in GO functional similarity score across correlation values**. Lines represent Anoxia dataset (solid line), Reoxygenation dataset (dashed line) and Alpha dataset (dotted line).

Functional similarity scores for the three datasets are displayed in Figure 1. Changes in scores across correlation values were similar for all datasets, and the lack of GO term relationship for negative correlations is striking. Because of this, the GO threshold was defined by the curve for positive correlations. Biological relationship begins to increase sharply above a correlation value of 0.80, and this produced the GO thresholds in Table 1.

**Table 1:** Estimated threshold for each method by dataset, with methods sorted by the sum of absolute deviations from the GO functional similarity threshold.

Method	Anoxia	Reoxygenation	Alpha	Absolute deviations from GO threshold
GO Functional Similarity	0.97	0.92	0.85	

Spectral Clustering	0.93	**0.97**^a^	**0.89**	0.04+0.05+0.04 = 0.13

Maximal Clique-2	0.90	0.91	0.74	0.07+0.01+0.11 = 0.19

Power	0.88	**0.94**	**0.96**	0.09+0.02+0.11 = 0.22

Bonferroni adjustment	0.85	**0.93**	**0.95**	0.12+0.01+0.10 = 0.23

Control-Spot	0.93	0.83	0.70	0.04+0.09+0.15 = 0.28

Maximal Clique-3	0.87	0.89	0.60	0.10+0.03+0.25 = 0.38

Top 1 Percent	0.81	0.81	0.72	0.16+0.11+0.13 = 0.40

^a^Thresholds above the GO functional similarity threshold are in bold.

Estimated thresholds obtained by each method are listed in Table 1 for the three datasets. If estimated threshold is higher than the biological threshold, false negatives will occur, because data reduction by the higher threshold will remove real relationships. Conversely, using a threshold below the biological threshold will create false positives, and relationships that are not real would be included in the network. In discovery-based settings, false positives are more acceptable, as they can be removed with further validation. Thus methods that estimate a lower threshold are preferred. Maximal Clique-2 and Spectral Clustering performed better than the other methods, based on summed absolute deviations from GO threshold (Table 1). Maximal Clique-2 was further from the GO threshold, but might be preferred since it never exceeded that threshold.

The estimated threshold derived for selected methods for each dataset is compared to bootstrap distributions in Table 2. The best methods from above, Maximal Clique-2 and Spectral Clustering, and two other methods for comparative purposes were chosen for this analysis. The bootstrap mean was never less than the estimated threshold, and occasionally was two standard deviations above. This upward bias in correlation is expected, as each time period had a limited number of arrays, making it likely that the identical array would be resampled. However, Maximal Clique and Spectral Clustering methods showed more resistance to this bias. The bootstrap standard deviation measures ability of the methods to produce similar threshold estimates from randomized arrays. Again the network-based methods showed the lowest standard deviations, and highest stability. All methods showed poorest performance with the Alpha dataset, possibly due to its unreplicated design. This makes it less likely that all time levels would be represented in the bootstrap samples, whereas the other datasets had glucose and galactose biological replicates.

**Table 2:** Summary of bootstrap results compares the estimated threshold with the bootstrap distribution for the four selected methods.

Method	Dataset	Estimated Threshold	Bootstrap Mean	**Difference**^**a**^	Bootstrap Standard Deviation
**Maximal Clique-2**	Anoxia	0.90	0.91	-0.01	0.015
	Reoxy	0.91	0.93	-0.02	0.009
	Alpha	0.74	0.78	-0.04	0.057

**Spectral Clustering**	Anoxia	0.93	0.95	-0.02	0.012
	Reoxy	0.97	0.97	0.00	0.011
	Alpha	0.89	**0.95	-0.06	0.017

**Top 1 Percent**	Anoxia	0.81	0.83	-0.02	0.011
	Reoxy	0.81	0.84	-0.03	0.016
	Alpha	0.72	**0.79	-0.07	0.027

**Control Spot**	Anoxia	0.93	0.95	-0.02	0.015
	Reoxy	0.83	**0.90	-0.07	0.034
	Alpha	0.70	**0.82	-0.12	0.043

^a ^Estimated threshold minus bootstrap mean.

** Estimated threshold is more than 2 std. deviations from bootstrap mean.

## Discussion

The two network-based methods, Maximal Clique-2 and Spectral Clustering, performed very well in terms of bootstrap stability and biological validity. Though Maximal Clique-2 method gave thresholds close to the biological threshold, and always below, the method had slightly higher bootstrap standard deviations. The robustness of the Maximal Clique-2 algorithm could be enhanced by exclusion of smaller cliques in the graph, for example cliques of size 3. Spectral Clustering thresholds were on average closer to biological thresholds, but too often exceeded it. However, if all thresholds for Spectral Clustering were lowered by 0.05, it would have been clearly the best method. Further fine-tuning of the parameters in the algorithm (size of sliding window, different tolerance levels for cluster formation) may improve the method's validity. In a recent paper, Almendral and Díaz-Guilera [21] documented the sensitivity of the non-zero eigenvalue to network changes. All methods had subjective settings, and further work on many more species and experiments would be needed to establish best choices.

The results from this study complement the work of Zhang and Horvath [1] which concluded that thresholds based on the scale-free topology - the formation of hubs and densely-connected sub-graphs - produced more robust results. The statistically-based methods studied here are directly dependent on the correlation distribution and thus were unable to capture biological relationships. Although the Control-Spot method is based on logical reasoning, the high correlation of control spots with other genes on the arrays weakened the method's validity. The Top 1% Correlations method is arbitrary, and failed to capture biological relationships. Statistical considerations used for the Power and Bonferroni methods were also not able to identify biological relationships, reflecting the well-known discrepancy between biological and statistical significance. Experiments that are small will produce thresholds that are too high, while large experiments will give excessively low thresholds, even though the biological relationships are the same.

The GO similarity measure of biological validity we have used, however, is by no means perfect and is just one way of quantifying biological information. Khatri and Draghici [22] have listed limitations of GO in detail. We also found low GO scores at high negative correlations as compared to the high GO score associated with high positive correlations for all three datasets. The drop in GO score at high negative correlations could be due to several reasons, for example experimental and analytical limitations to detect biologically negative correlations among genes, and limited gene annotations [11]. As the quantification of biological information in data gets more precise, the selection of thresholds should become easier. In fact, note that a method like the GO threshold used here would be a logical choice if GO information were complete and accurate.

## Competing interests

The authors declare that they have no competing interests.

## Authors' contributions

BRB wrote code for the analyses, summarized results, and drafted the paper. All authors were involved in study design, and read and approved the final manuscript.

## Supplementary Material

Additional file 1 **Methodology for Threshold Estimation**. Details on the six threshold estimation methods are presented in a computationally oriented manner. Click here for file

## References

[B1] Zhang B Horvath S A general framework for weighted gene co-expression network analysis Stat Appl Genet Mol Biol 2005 4 16646834 10.2202/1544-6115.1128

[B2] Butte AJ Tamayo P Slonim D Golub TR Kohane IS Discovering functional relationships between RNA expression and chemotherapeutic susceptibility using relevance networks Proc Natl Acad Sci USA 2000 97 22 12182 12186 10.1073/pnas.220392197 11027309 PMC17315

[B3] Voy BH Scharff JA Perkins AD Saxton AM Borate B Chesler EJ Branstetter LK Langston MA Extracting gene networks for low-dose radiation using graph theoretical algorithms PLoS Comput Biol 2006 2 7 e89 10.1371/journal.pcbi.0020089 16854212 PMC1513268

[B4] Yan X Mehan MR Huang Y Waterman MS Yu PS Zhou XJ A graph-based approach to systematically reconstruct human transcriptional regulatory modules Bioinformatics 2007 23 13 i577 586 10.1093/bioinformatics/btm227 17646346

[B5] Freeman TC Goldovsky L Brosch M van Dongen S Maziere P Grocock RJ Freilich S Thornton J Enright AJ Construction, visualisation, and clustering of transcription networks from microarray expression data PLoS Comput Biol 2007 3 10 2032 2042 10.1371/journal.pcbi.0030206 17967053 PMC2041979

[B6] Baldwin NE Chesler EJ Kirov S Langston MA Snoddy JR Williams RW Zhang B Computational, integrative, and comparative methods for the elucidation of genetic coexpression networks J Biomed Biotechnol 2005 2005 2 172 180 10.1155/JBB.2005.172 16046823 PMC1184052

[B7] Butte AJ Kohane IS Mutual information relevance networks: functional genomic clustering using pairwise entropy measurements Pac Symp Biocomput 2000 418 429 10902190 10.1142/9789814447331_0040

[B8] Quackenbush J Genomics. Microarrays--guilt by association Science 2003 302 5643 240 241 10.1126/science.1090887 14551426

[B9] Sanoudou D Haslett JN Kho AT Guo S Gazda HT Greenberg SA Lidov HG Kohane IS Kunkel LM Beggs AH Expression profiling reveals altered satellite cell numbers and glycolytic enzyme transcription in nemaline myopathy muscle Proc Natl Acad Sci USA 2003 100 8 4666 4671 10.1073/pnas.0330960100 12677001 PMC153613

[B10] Moriyama M Hoshida Y Otsuka M Nishimura S Kato N Goto T Taniguchi H Shiratori Y Seki N Omata M Relevance network between chemosensitivity and transcriptome in human hepatoma cells Mol Cancer Ther 2003 2 2 199 205 12589037

[B11] Lee HK Hsu AK Sajdak J Qin J Pavlidis P Coexpression analysis of human genes across many microarray data sets Genome Res 2004 14 6 1085 1094 10.1101/gr.1910904 15173114 PMC419787

[B12] Langston MA Perkins AD Saxton AM Scharff JA Voy BH Innovative Computational Methods For Transcriptomic Data Analysis: A Case Study in the Use Of FPT For Practical Algorithm Design and Implementation ACM symposium on Applied Computing: 2006; Dijon, France 2006

[B13] Palla G Derenyi I Farkas I Vicsek T Uncovering the overlapping community structure of complex networks in nature and society Nature 2005 435 7043 814 818 10.1038/nature03607 15944704

[B14] Perkins AD Langston MA Threshold selection in gene co-expression networks using spectral graph theory techniques BMC Bioinformatics 2009 10 Suppl 11 S4 10.1186/1471-2105-10-S11-S4 19811688 PMC3152776

[B15] Lai LC Kosorukoff AL Burke PV Kwast KE Metabolic-state-dependent remodeling of the transcriptome in response to anoxia and subsequent reoxygenation in Saccharomyces cerevisiae Eukaryot Cell 2006 5 9 1468 1489 10.1128/EC.00107-06 16963631 PMC1563586

[B16] Spellman PT Sherlock G Zhang MQ Iyer VR Anders K Eisen MB Brown PO Botstein D Futcher B Comprehensive identification of cell cycle-regulated genes of the yeast Saccharomyces cerevisiae by microarray hybridization Mol Biol Cell 1998 9 12 3273 3297 9843569 10.1091/mbc.9.12.3273 PMC25624

[B17] Zhang Y Abu-Khzam FN Baldwin NE Chesler EJ Langston MA Samatova NF Genome-scale computational approaches to memory-intensive applications in systems biology Supercomputing 2005 Proceedings of the ACM/IEEE SC Conference: 2005 2005 12

[B18] Chung FRK Spectral Graph Theory 1994 American Mathematical Society

[B19] Ding CHQ He X Zha H A spectral method to separate disconnected and nearly disconnected Web graph components Proceedings of the Seventh ACM SIGKDD International Conference on Knowledge Discovery and Data Mining. San Francisco, California 2001 275 280 http://ranger.uta.edu/~chqding/papers/kdd3a.ps full_text

[B20] Politis DN The impact of bootstrap methods on time series analysis Statistical Science 2003 18 2 219 230 10.1214/ss/1063994977

[B21] Almendral JA Diaz-Guilera A Dynamical and spectral properties of complex networks New Journal of Physics 2007 9 187 10.1088/1367-2630/9/6/187

[B22] Khatri P Draghici S Ontological analysis of gene expression data: current tools, limitations, and open problems Bioinformatics 2005 21 18 3587 3595 10.1093/bioinformatics/bti565 15994189 PMC2435250

