# Supplementary material for: Comparison of threshold selection methods for microarray gene co-expression matrices
Source: BMC Res Notes. 2009 Dec 2;2:240. doi: 10.1186/1756-0500-2-240 (PMC2794870; doi:10.1186/1756-0500-2-240)
Supplement: Additional file 1 — Methodology for Threshold Estimation. Details on the six threshold estimation methods are presented in a computationally oriented manner. [file 1756-0500-2-240-S1.PDF]

Additional File 1

Methods for Threshold Estimation

Expression data from each of the three datasets were filtered for control spots and unexpressed genes. Pearson correlations among all remaining genes within a dataset were calculated using the Datagen software (from the Langston lab). This software also averaged technical replicate arrays, as correlations among technical replicates would create positive bias.

1) Maximal Clique-2 and Maximal Clique-3. Clique software from Langston's lab was used.

Start with a threshold of r=0.99, and create a graph from the correlation matrix. Run clique software on this graph to get the number of maximal cliques. Repeat, incrementally decreasing the correlation threshold in steps of 0.01. When number of maximal cliques is two times higher than with the previous threshold, the current correlation value is taken as the final Maximal Clique-2 threshold choice. Similarly, the Maximal Clique-3 threshold is chosen where number of maximal cliques is three times higher than the previous correlation step.

At high correlation thresholds, the graph can be sparse, giving low numbers of maximal cliques. This could produce a false threshold, as the number of maximal cliques will increase two or three fold from such low initial numbers. Therefore a threshold was not chosen until number of maximal cliques exceeded 50,000.

2) Spectral graph. MATLAB 7.0 was used for matrix calculations.

Using a threshold of r=0.99, create a graph from the correlation matrix, with edges between genes only if their correlation exceeds the threshold. This graph is represented as an adjacency matrix (A) and a degree matrix (D). Using spectral graph theory (Ding et al. 2001), the Laplacian matrix is A-D. Compute eigenvalues and eigenvectors for the Laplacian matrix. Take the eigenvector associated with the smallest non-zero eigenvalue, and count numbers of clusters. This is done with a sliding window 10 elements wide, and a new cluster is identified when the highest minus lowest value in the window exceeded the median value+ std. dev./2.

Repeat the above for incrementally smaller correlations, in steps of 0.01, until a peak in the number of identified clusters is found. The correlation which produces the maximum number of clusters is the threshold.

3) Control Spot

Correlations of control spots with all other genes on the array were calculated, creating a null distribution of correlation values. Absolute values of the correlations were ranked to find the 99th percentile, and that correlation value was chosen as the threshold. The concept used here is the control spot correlations should represent noise, so setting a threshold that excludes 99% of the noise may be effective.

4) Top 1%
Correlation values among all genes, with control spots and unexpressed genes filtered out, were sorted to identify the 99th percentile. The associated correlation value produced the threshold estimate. Note that the control spot method uses a different subset of correlations (only with control spots), whereas this method uses all correlations among genes. This method is based on the (arbitrary) assumption that only the highest 1% of the correlation values will contain biological relationships.

5) Bonferroni p-value. SAS 9.1 software (Cary, NC) was used for these calculations. A p-value for every correlation among genes, testing if the correlation was zero using Fisher's z-transformation, $z = 0.5 * \ln[(1+r)/(1-r)]$, to create a standard normal hypothesis test. P-values from these tests are sorted to find the critical Bonferroni p-value, 0.05/number of correlations. The threshold is the correlation associated with that critical p-value.

Computations can be reduced if necessary, by taking the critical Bonferroni p-value, obtaining the associated z-statistic, then back solving for the threshold correlation using Fisher's z-transformation equation.

This threshold will remove any correlations that are statistically equal to zero. FDR and q-value adjustments to raw p-values were considered as an alternative to Bonferroni, but their distributions were almost the same as the raw p-values. This was probably due to the millions of p-values being adjusted for.

6) Statistical power . PASS software (http://www.ncss.com/pass.html) was used. For the hypothesis test of Ho: correlation=0, start with an alternate hypothesis that r=0.99. Use Fisher's z-transformation, $z = 0.5 * \ln[(1+r)/(1-r)]$, to create a standard normal hypothesis test. Using the number of observations for a gene, and a Bonferroni protected Type I error rate of 0.05/number of correlations, compute the statistical power. Iterate through other alternate hypothesis values for r (a bisection search will reduce computation) until the r that produces 80% power is found. This r is the chosen threshold. This approach sets the threshold where there is a reasonable chance (80%) of correctly identifying correlations that are different from zero, and hopefully are biologically "real". This differs from Bonferroni p-value (method 5) which focuses on the null hypothesis and Type I error rate, as opposed to the alternate hypothesis and statistical power.

References

Ding CHQ, He X, Zha H: **A spectral method to separate disconnected and nearly disconnected Web graph components**. In: *Proceedings of the Seventh ACM SIGKDD International Conference on Knowledge Discovery and Data Mining.* San Francisco, California; 2001: 275 - 280.
